# Notice

**Published:** 1889-07

**Authors:** Robert P. Porter

**Affiliations:** Superintendent of Census; Department of the Interior, Census Office, Washington, D. C.


### NOTICE.

---

DEPARTMENT OF THE INTERIOR,
CENSUS OFFICE,
Washington, D. C., May 1, 1889.

*To the Medical Profession:*

The various medical associations and the medical profession will be glad to learn that Dr. John S. Billings, Surgeon U. S. Army, has consented to take charge of the Report on Mortality and Vital Statistics of the United States as returned by the eleventh census.

As the United States has no system of registration of vital statistics, such as is relied upon by other civilized nations for the purpose of ascertaining the actual movement of population, our census affords the only opportunity of obtaining near an approximate estimate of the birth and death rates of much the larger part of the country, which is entirely unprovided with any satisfactory system of State and municipal registration.

In view of this, the census office, during the month of May this year, will issue to the medical profession throughout the country "Physicians' Registers" for the purpose of obtaining more accurate returns of death than it is possible for the enumerators to make. It is earnestly hoped that physicians in every part of the country will co-operate with the census office in this important work. The record should be kept from June 1, 1889, to May 31, 1890. Nearly 26,000 of these registration books were filled up and returned to the office in 1880, and nearly all of them used for statistical purposes. It is hoped that double this number will be obtained for the eleventh census.

Physicians not receiving Registers can obtain them by sending their names and addresses to the census office, and, with the Register, an official envelope which requires no stamp will be provided for their return to Washington.

If all medical and surgical practitioners throughout the country will lend their aid, the mortality and vital statistics of the eleventh census will be more comprehensive and complete than they have ever been. Every physician should take a personal pride in having this report as full and accurate as it is possible to make it.

It is hereby promised that all information obtained through this source shall be held strictly confidential.

ROBERT P. PORTER,
*Superintendent of Census.*

### ITEMS.

TETANUS NEONATORUM (?) AND SEPTIC PERITONITIS PROBABLY DUE TO CIRCUMCISION.—Dr. Oscar A. Fliesburg reports the following case:—December 25th, the author was called in to see an infant, aged eleven days. On examination he found the jaws firmly set, lips somewhat drawn, the child crying and whining, unable to take the breast or even swallow anything, except by letting it slowly trickle down the mouth and throat, extremities limp, but the legs drawn upward to abdomen, eyes normal in appearance, retention of urine for eighteen hours, bowels opened previous night, very small amount and greenish black in color.

The author found that the child had been circumcised on December 22d, and that it had first shown symptoms of illness on the following day. The penis was found to be in a very filthy condition, the wound not healing, but suppurating, the glans penis somewhat swollen, odor of decomposing urine emitted from the diaper, the whole penis and scrotum imbedded in lycopodium, as is the habit of the Jewish rabbi to leave the organ after performing the religious rite. Temperature 99.4° Fahr.; respiration hurried, somewhat sighing.

The author at once cleansed the penis antiseptically and as much as possible disinfected the wound and catheterized the patient, but found the urine so thick and of so small an amount that only a few drops of whitish, very thick fluid escaped. The child lived a couple of days, or from the 25th to the 27th, the symptoms changing very little. On the 25th he urinated twice, and the same on the 26th; on the latter date symptoms of peritonitis became marked, tympanites, pain on pressure, sighing respiration, drawn up extremities, etc.

The writer bases his diagnosis on the following:

First, the rabbi had inoculated the wound through the practice of the Jews, when performing the "Mosaic rite," of sucking the phallus to stop the bleeding after completing the operation.

The author does not think it a case of idiopathic tetanus neonatorum:

1. Because the trismus did not show itself until the ninth day. 2. The onset was gradual, without convulsions or rigors. 3. The symptoms were different, beginning with tetanic locking of jaw; no vomiting, or very little; no coma, at least not until late in the course of the disease. 4. The peritonitic symptoms were unquestionably due to septic absorption. 5. The writer does not believe in trismus neonatorum without traumatic origin.—*Northwestern Lancet*, February 19th, 1889.

SUPPRESSION OF URINE SEVEN DAYS IN A CHILD TWO YEARS OLD.—Dr. E. P. Benardy, in a paper read before the Obstetrical

Society, Philadelphia, gave the details of an interesting case: Was called in to see the child August 17, 1888. He had been ailing for a few days, and was suffering from nausea and vomiting; bowels loose; tongue clean, white and flabby; skin white, eyes dull, pulse quick, but no fever. Pepsin mixture was ordered. The next day stomach was irritable, and he could not retain anything on his stomach; pulse full and quick, but no fever; was informed that he had not passed urine for some time, ordered xx gtts. spt. æther nit. in warm water every hour or two. The following day condition about the same, but passed a quarter teaspoonful pure blood from the penis. Examination over region of bladder showed no indication of fluid. Ordered warm digitalis and flaxseed poultice applied on region of kidneys—a bitart. potassa mixture, with infusion digitalis internally. Next day showed no improvement, and twitchy movements of the hips and lower extremities was easily started.

On August 21, 22 and 23 the symptoms increased to an alarming extent, the skin being burning hot, and no urine being passed, close examination over bladder showing it to be empty. Six dry cups were applied over region of kidneys, and five hours afterward he passed a large quantity of clear urine.

August 25, all nervous symptoms abating, he passed urine of a light yellowish color, and a general improvement was manifest. This continued, and the patient was discharged cured, September 5, 1888.

The case was interesting on account of its rarity, even partial suppression of urine resulting in coma, convulsions and death.—*June Am. Med. Ass'n.*

THE following new appointments have been made at the New York Polyclinic: Dr. Thomas B. Pooley, Surgeon in Chief of the New Amsterdam Eye and Ear Hospital; Ophthalmic Surgeon to the Sheltering Arms; consulting Ophthalmologist to St. Bartholomew's Hospital; Professor of Ophthalmology. Dr. B. Sachs, consulting Neurologist to the Montifiore, home for Chronic Invalids; Professor of Neurology. Dr. L. Emmett Holt, visiting Physician to the New York Infant Asylum; consulting Physician to the Hospital for Ruptured and Crippled; Professor of Diseases of Children. Dr. August Seibert, Physician to the Children's Department to the German Dispensary; Professor of Diseases of Children. Dr. A. Marion Sims, Gynecologist to St. Elizabeth's Hospital and New York Infant Asylum; Professor of Gynecology. Dr. Wm. H. Flubrer, Surgeon to Mt. Sinai and Bellevue Hospital; Professor of Genito-Urinary Surgery. The Polyclinic has increased its Hospital facilities by the purchase of a large building immediately adjoining its original property, and after making necessary changes will furnish and have it open by September 16, when the regular session will commence.

THE LOCAL ACTION OF HYDRASTIS CANADENSIS.—Felsenberg, in the *Wiener med. Blätter*, November 29, 1888, writes a laudatory notice of this drug, as regards its influence upon the blood-vessels of mucous membranes in gynecological cases, and in diseases of the mouth, nose, and similar parts where there is congestion. Felsenburg, after giving the credit to American physicians of having introduced the drug, then goes on to tell us that it has been found to be not only an astringent, but, in addition, to possess local anæsthetic properties. In relation to his own experience with the drug, he mentions his success in the treatment of cases of chronic pharyngitis combined with enlargement of the tonsils. In every instance he painted the fluid extract over the diseased mucous membrane, thoroughly covering all portions that were inflamed. The application was not found to be exceedingly disagreeable, and was very effective. Exceedingly good results were also reached by application to chronic inflammation of the vagina.—*Ex.*

IGNIPUNCTURE OF THE TONSILS.—Dr. Wilhelm Roth, of Fluntern, finds that in order to reduce the size of the tonsils without risk of troublesome hemorrhage, which is not uncommon, especially in young subjects, the best plan is to employ ignipuncture, as has been recommended by Krishaber and more recently by Verneuil. The tonsils and neighboring parts are first brushed over with a 10 to 20 per cent. solution of cocaine. The finest point of the thermocautery, heated to redness, is then inserted to a depth of about five millimeters in three or four spots, a few millimeters apart from one another on the tonsils. The instrument is not allowed to remain more than one or two seconds in the tissue. The whole operation, including both tonsils, can be performed in a very few minutes without any bleeding, and with scarcely any pain. It must be repeated four or five times at intervals of two or three days, and this is usually sufficient to cause the tonsils to return to their ordinary condition.—*Lancet.*

A PHYSICIAN who understands human nature, plays with the baby, makes friends with the children and listens to the woes of the good wife and mother, is the fellow to whom the master of the house most cheerfully pays the largest bills. It's the comfort, the consolation, that mark the broad line between an unsuccessful and a popular physician.—*Ex.*

ANTIPYRIN AS A HÆMOSTATIC.—Antipyrin has been lauded as a hæmostatic. As such it acts by preventing the formation of fibrin so thoroughly and effectually that there is less resistance to the flow through capillary blood-vessels in the vicinity than into the atmosphere, and more attraction.

The following changes have been made in the faculty of the Medico-Chirurgical College, of Philadelphia: Frank Woodbury, A. M., M. D., honorary professor of clinical medicine; William B. Atkinson, A. M., M. D., honorary professor of sanitary science and pædiactrics; John V. Shoemaker, A. M., M. D., professor of materia medica, pharmacology, therapeutics and clinical medicine; James M. Anders, Ph. D., M. D., professor of hygiene and clinical diseases of children.

THERE were two errors in the last issue of the JOURNAL which escaped our notice and to which we wish to call attention. In the article by Dr. W. F. Westmoreland, Jr., it should have read: "Reduction of sub-caracoid by Kocher's method." In the report of the Atlanta Society of Medicine, Dr. McRae was quoted as advising the immersion of instruments in 1–1000 bichloride solution, when it should have been 1–30 carbolic acid solution.

WE call the attention of the profession to the letter of Robert L. Porter, Superintendent of the Census, soliciting the co-operation of medical men in obtaining an accurate report on mortality and vital statistics in the United States. Dr. John S. Billings, Surgeon of the U. S. Army, has consented to take charge of the report, and we hope that the physicians throughout the country will respond promptly to the request.

THE RELATIVE VALUE OF THE BROMIDES.—Every 10 grains of sodium bromide contains 7.76 grains of bromine, and every 10 grains of potassium bromide contain 6.72 grains of bromine; so that in order to prescribe the same weight of bromine, we must give instead of 10 grains of bromide of potassium, only 8.6 grains of bromide of sodium. So also with the iodide of potassium, a 10-grain dose is represented by a 9-grain dose of iodide of sodium. —*British Medical Journal.*

